# Identification of a vacuolar proton channel that triggers the bioluminescent flash in dinoflagellates

**DOI:** 10.1371/journal.pone.0171594

**Published:** 2017-02-08

**Authors:** Juan D. Rodriguez, Saddef Haq, Tsvetan Bachvaroff, Kristine F. Nowak, Scott J. Nowak, Deri Morgan, Vladimir V. Cherny, Maredith M. Sapp, Steven Bernstein, Andrew Bolt, Thomas E. DeCoursey, Allen R. Place, Susan M. E. Smith

**Affiliations:** 1 Department of Molecular and Cellular Biology, Kennesaw State University, Kennesaw, Georgia, United States of America; 2 Institute of Marine and Environmental Technology, University of Maryland Center for Environmental Science, Baltimore, Maryland, United States of America; 3 Department of Molecular Biophysics and Physiology, Rush University, Chicago, Illinois, United States of America; University of South Florida, UNITED STATES

## Abstract

In 1972, J. Woodland Hastings and colleagues predicted the existence of a proton selective channel (H_V_1) that opens in response to depolarizing voltage across the vacuole membrane of bioluminescent dinoflagellates and conducts protons into specialized luminescence compartments (scintillons), thereby causing a pH drop that triggers light emission. H_V_1 channels were subsequently identified and demonstrated to have important functions in a multitude of eukaryotic cells. Here we report a predicted protein from *Lingulodinium polyedrum* that displays hallmark properties of bona fide H_V_1, including time-dependent opening with depolarization, perfect proton selectivity, and characteristic ΔpH dependent gating. Western blotting and fluorescence confocal microscopy of isolated *L*. *polyedrum* scintillons immunostained with antibody to LpH_V_1 confirm LpH_V_1’s predicted organellar location. Proteomics analysis demonstrates that isolated scintillon preparations contain peptides that map to LpH_V_1. Finally, Zn^2+^ inhibits both LpH_V_1 proton current and the acid-induced flash in isolated scintillons. These results implicate LpH_V_1 as the voltage gated proton channel that triggers bioluminescence in *L*. *polyedrum*, confirming Hastings’ hypothesis. The same channel likely mediates the action potential that communicates the signal along the tonoplast to the scintillon.

## Introduction

The first postulation that a depolarization-activated, proton selective channel (H_V_1) should exist was published in 1972 by J. Woodland Hastings and colleagues [1]. A decade later, Thomas and Meech reported the first voltage-gated proton conductance to be identified by voltage-clamp studies, in snail neurons [2]. Subsequent electrophysiological studies have elucidated the defining characteristics of a family of voltage-gated proton-selective channels, H_V_1, which have been found in amphibia [3], rat [4], human [5–8], insects [9], and both multicellular [10] and unicellular marine species [11,12]. H_V_1 are exquisitely selective for protons [13], resulting from a critical Asp residue in the S1 transmembrane helix [12,14] that is thought to interact with an arginine in S4 [15,16]. H_V_1 have a single-channel conductance 10^3^ smaller than most ion channels [17], reflecting the 10^6^ lower concentration of permeant ions. They open with depolarization but their voltage-dependence is strongly influenced by both external and internal pH, pH_o_ and pH_i_ [18], such that a one unit change in either pH_o_ or pH_i_ (or in the pH gradient ΔpH = pH_o_—pH_i_) shifts the *g*_H_-*V* relationship by 40 mV [19]. Predicted H_V_1 genes are nearly ubiquitous in eukaryotic genomes and the protein has multiple demonstrated functions in various eukaryotic cells [20].

Dinoflagellate bioluminescence is a striking phenomenon, producing brilliant blue flashes in the ocean water when the organisms are stimulated mechanically [21] in the form of shear stress [22,23]. The biochemical basis of dinoflagellate light production was elucidated in seminal work over several decades by Hastings and colleagues, who focused mainly on *Lingulodinium polyedrum* (formerly *Gonyaulax polyedra*). This work established that the light originates in organelles called scintillons arising from evaginations of the central vacuole membrane [24–28]. Scintillons contain a breakdown product of chlorophyll [29] called luciferin that is bound to luciferin binding protein (LBP) at pH > 7, while at pH < 7 LBP releases luciferin, thereby enabling it to interact with the enzyme luciferase (LCF) that catalyzes the reaction with oxygen and luciferin to produce light [1,24,26,30–32]. Low pH activates the LCF in bioluminescent dinoflagellates [26,33] providing a second mechanism by which protons trigger the flash.

Fogel and Hastings [1] reasoned that a hypothetical voltage-sensitive proton channel should respond to a stimulus-induced depolarization of the *L*. *polyedrum* vacuole membrane [34], and that such a channel would transport protons from the acidic vacuole into the scintillons, thus providing the pH change that triggers bioluminescence. H_V_1 proton channels have been reported in several non-bioluminescent unicellular marine species [11,12] where they function in calcium fixation (coccoliths) and possibly in feeding (dinoflagellates). Here we identify a bona fide H_V_1 in the bioluminescent species studied by Hastings and colleagues, *L*. *polyedrum*, and demonstrate its scintillon localization. In addition to confirming a longstanding prediction we authenticate a unique mode for H_V_1 in the vacuole membrane as the control step in the signal transduction pathway that leads to dinoflagellate bioluminescence.

## Results

Sequence similarity searches of RNA-seq assemblies [35,36] from the bioluminescent organism *L*. *polyedrum* revealed a sequence (gi: 346282507) with the signature sequence pattern [12] that has proven to be diagnostic of H_V_1 (longest open reading frame from this assembled transcript is shown in S3 Table.) cDNA libraries prepared from *L*. *polyedrum* populations sampled at mid-light and mid-dark and probed with PCR primers designed using the RNA-seq data produced PCR products with expected sizes from this putative H_V_1 (S1 Table). qRT-PCR shows that the RNA for the putative H_V_1 was expressed in *L*. *polyedrum* (data not shown).

### LpH_V_1 is a bona fide voltage-gated proton channel

**Fig 1:**
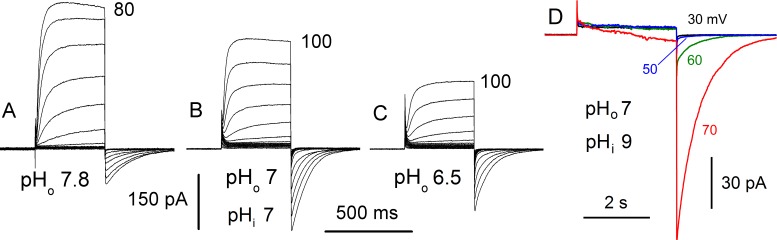
LpH_V_1 is a voltage gated proton channel. (A-C) Families of whole-cell proton currents at different pH_o_ in a cell transfected with LpH_V_1, with pH_i_ 7.0. Voltage pulses were applied from a holding potential of -60 mV (A, B), or -40 mV (C), in 10 mV increments up to the voltage indicated. (D) Inward H^+^ currents can be seen with large inward pH gradients. Currents are shown during pulses to 30, 50, 60, and 70 mV as indicated, in an inside-out patch with pH_o_ 7 (pipette) and pH_i_ 9 (bath), according to the standard convention in which downward deflections indicate inward current flow. From the tail currents upon repolarization to the holding potential of -40 mV, it is clear that the *g*_H_ was already activated detectably by the pulse to 50 mV, with small inward current evident during the pulse to 60 mV, and larger inward current at 70 mV.

We ordered the synthesis of a mammalian codon-optimized gene (sequence shown in S3 Table) corresponding to the predicted *L*. *polyedrum* H_V_1 gene. When the gene was expressed in a human cell line (HEK-293) the gene product produced voltage- and time-dependent currents in voltage-clamped cells (Fig 1). Non-transfected HEK-293 cells sometimes had small native proton currents, but otherwise had no significant conductances that exhibited time dependent activation or tail currents under our recording conditions. The proton current during depolarizing voltage pulses turned on more rapidly than in mammalian species in which activation time constants may be seconds at room temperature [4,5,6]. LpH_V_1 current also differed from most species studied to date in turning on much more rapidly at more positive voltages (discussed below). Similar to H_V_1 in all other species, LpH_V_1 exhibited strong sensitivity to pH. As in the cell in Fig 1A–1C, when pH_o_ was decreased, the voltage range over which the *g*_H_ was activated shifted positively.

#### LpH_V_1 can produce inward current

In the vacuole or scintillon membrane, the proton channel is expected to be oriented with its external side facing the vacuole. In order to mediate the flash-triggering action potential in the vacuole membrane and enable H^+^ influx into the scintillon to activate luciferase, the channel *in situ* must be able to conduct inward current. At symmetrical pH, activation occurred well positive to 0 mV (e.g., Fig 1B) and thus only outward current was produced, as in all other species except for *K*. *veneficum* [12]. In fact, the average “threshold” voltage at which LpH_V_1 was clearly activated was 46 ± 1.8 mV (mean ± SEM, *n* = 38; 23 cells and 15 patches), well positive to +23 mV reported for a variety of native proton currents, mostly in mammalian cells, or to -10 to +10 mV in hH_V_1 (Table 3 in [20]). However, Fig 1D shows that when there was a large inward pH gradient (simulating the low pH vacuole and high pH cytoplasm that exist in *L*. *polyedrum*), inward current was activated. *In vivo*, LpH_V_1 is exposed to an enormous inward pH gradient (ΔpH is 3.5), because the flotation vacuole has very low pH 4.5 [28], compared with cytoplasmic pH ~8. HEK-293 cells did not tolerate such a large gradient, but with a moderate inward gradient (ΔpH 2.0), we observed inward H^+^ current (Fig 1D). Inward currents were detected in ten cells with 1.0 to 3.0 U gradients. Extrapolated to *in vivo* conditions, LpH_V_1 should conduct inward current when activated.

#### LpH_V_1 is proton selective

**Fig 2:**
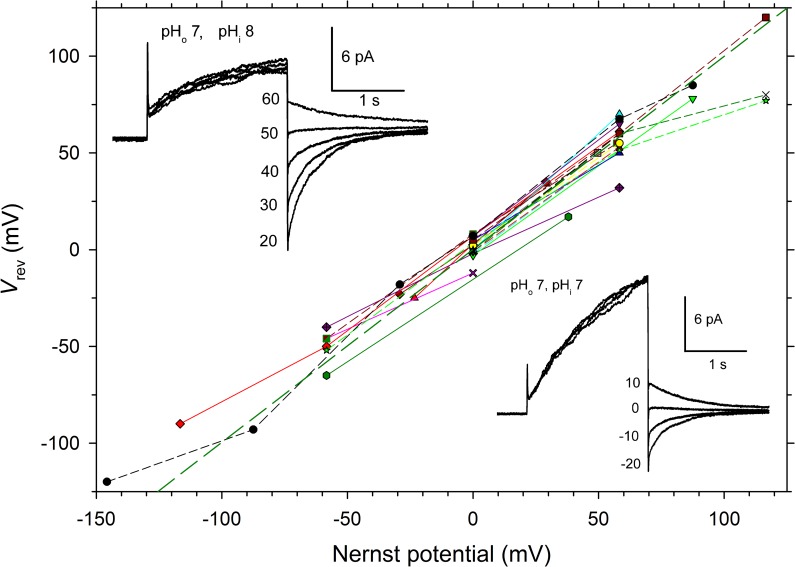
LpH_V_1 is a proton selective channel. The reversal potential (*V*_rev_) was measured, usually by tail currents (as shown in the examples in the insets), in both whole-cell (*n* = 10) and excised, inside-out patch configurations (*n* = 10) over a wide range of pH (pH_o_ 4.5–9.0; pH_i_ 4.5–10.0). Measurements at multiple pH in individual cells or patches are connected by lines. The heavy dashed green line indicates *V*_rev_ = *E*_H_, which would indicate perfect proton selectivity. Whole-cell data are plotted as triangles, diamonds, or hexagons, and pink Xs, connected by solid lines; other symbols are from inside-out patches, connected by dashed lines. Insets show tail current measurements from the same inside-out patch, with pH_o_ 7 in the pipette, and pH_i_ 8 or 7, as indicated, in the bath. *V*_rev_ shifts from -2 mV at pH_i_ 7 to 53 mV at pH_i_ 8, a change of 55 mV, near the Nernst expectation of 58 mV for perfect H^+^ selectivity.

Measurement of the reversal potential (*V*_rev_) of the current in cells expressing LpH_V_1 over a wide range of pH_o_ and pH_i_ confirmed that these currents were proton selective, because *V*_rev_ was close to the Nernst potential for H^+^ (*E*_H_) (dashed green line in Fig 2). When tetramethylammonium^+^ was replaced by other small cations *V*_rev_ did not change, confirming H^+^ selectivity. The mean change in *V*_rev_ when TMA^+^ in the bath was exchanged with Na^+^ or K^+^, respectively, was 0.4 ± 0.4 mV (mean ± SEM, *n* = 4) or 1.2 ± 1.8 mV (*n* = 4), after correction for the measured liquid junction potentials. The expressed gene product is clearly a highly proton-selective voltage-gated channel, so we named it LpH_V_1, and explored its similarities and differences from H_V_1 in other species.

#### LpH_V_1 exhibits ΔpH dependent gating

**Fig 3:**
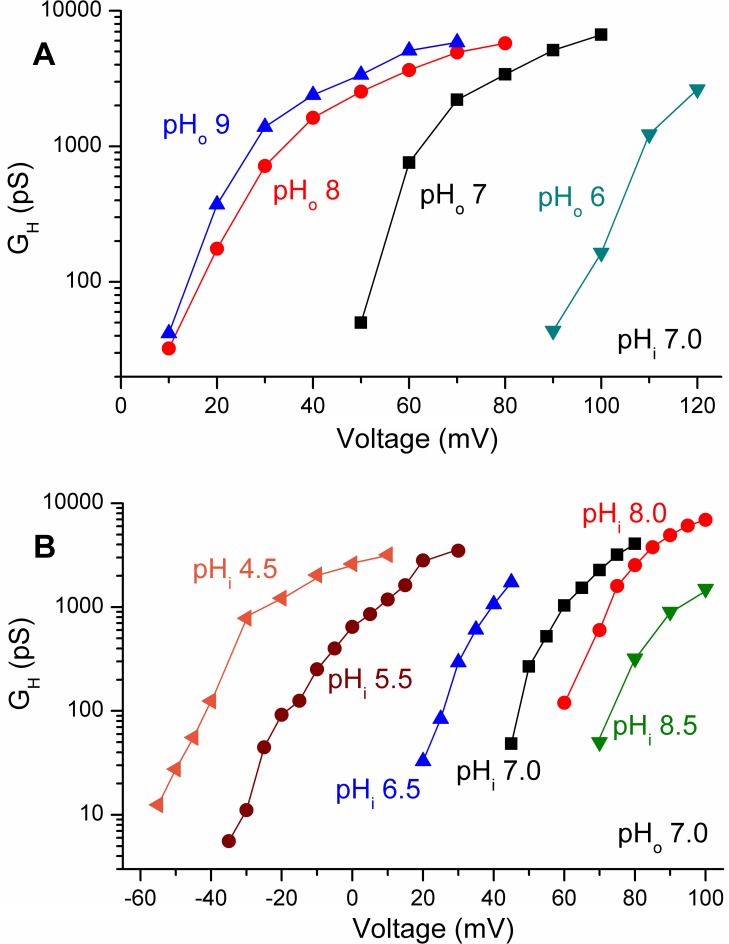
LpH_V_1 exhibits classical ΔpH dependent gating. The *g*_H_-*V* relationships calculated from LpH_V_1 currents in whole cell measurements (A) or inside-out patches (B) are strongly affected by pH_o_ or pH_i_, respectively. The proton conductance, *g*_H_, was usually calculated from the measured reversal potential, *V*_rev_, and the amplitude of a single rising exponential fitted to the current. In some cases, for example with test pulses near *V*_rev_, the amplitude of the tail current was used, after appropriate scaling.

In all species studied so far, H_V_1 exhibits ΔpH dependent gating, in which the voltage range of channel opening is defined by the pH gradient, ΔpH (pH_o_ - pH_i_). The position of the proton conductance-voltage, *g*_H_-*V*, relationship generally shifts 40 mV/unit change in either pH_o_ or pH_i_ (positively for increasing pH_i_ and negatively for increasing pH_o_) [13,18,19]. Fig 3 shows *g*_H_-*V* relationships in a cell studied in whole-cell configuration with changes in pH_o_ (A) and in an excised, inside-out patch of membrane in which pH_i_ was changed (B). It is evident from Fig 3A that increasing pH_o_ shifts the voltage dependence of LpH_V_1 opening negatively, as observed in H_V_1 from all species studied to date. It is further evident that there was little shift between pH_o_ 8 and pH_o_ 9, indicating that this effect saturates at high pH_o_, as reported recently in human H_V_1, hH_V_1, kH_V_1, and EhH_V_1 [37]. The data in Fig 3B show that changing pH_i_ similarly shifts the *g*_H_-*V* relationship, with decreasing pH_i_ shifting the curve negatively, as occurs in all other H_V_1.

**Fig 4:**
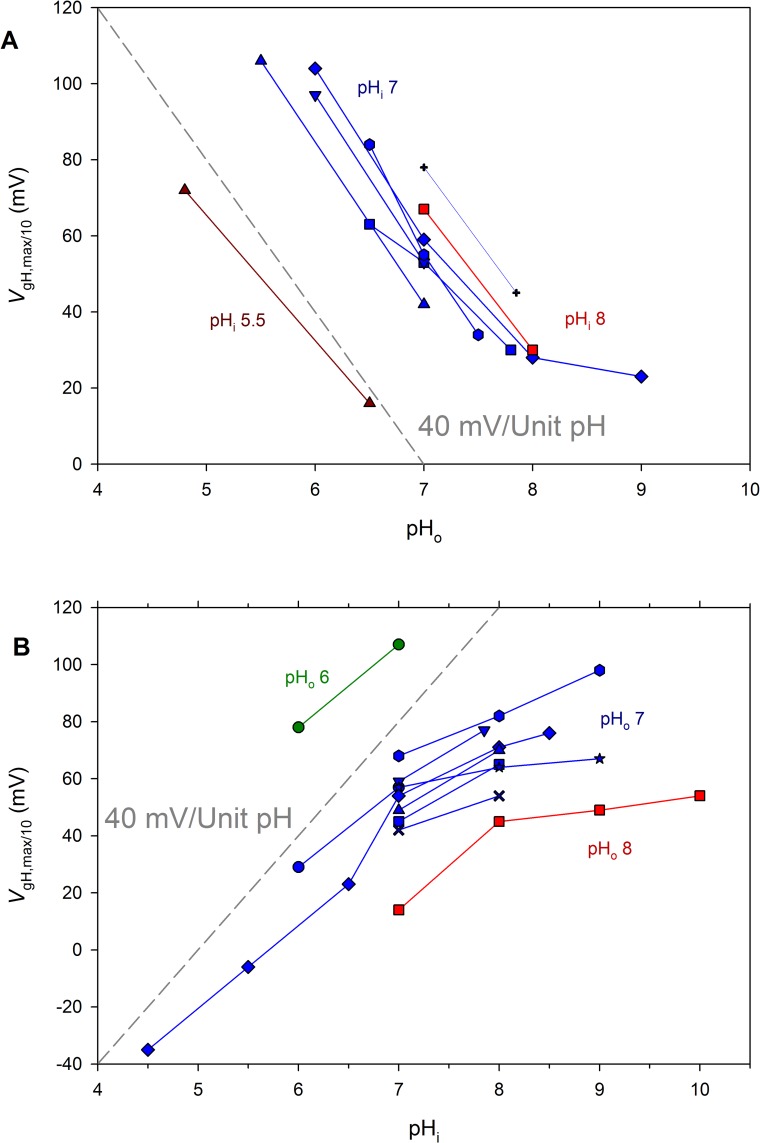
LpH_V_1 exhibits ΔpH dependent gating. The position of the *g*_H_-*V* relationship was established from *g*_H_-*V* relationship plots by measuring the voltage at which *g*_H_ was 10% of *g*_H,max_. Measurements in individual cells or patches at several pH are connected by lines. Color coding indicates pH_i_ for whole cell measurements (A) or pH_o_ for inside-out patch measurements (B). As a reference, the arbitrarily positioned dashed line in each panel shows the slope that corresponds to a shift of 40 mV/unit change in either pH_o_ or pH_i_. Except at high pH_o_ or pH_i_, the data are roughly parallel to the reference lines, indicating a slope of 40 mV/unit pH. The slope decreases at pH>8, indicating saturation of ΔpH dependence.

The effects of pH_o_ and pH_i_ on the position of the *g*_H_-*V* relationship were assessed quantitatively by plotting the voltage at which the *g*_H_ was 10% of its maximal value, *g*_H,max_, as a function of pH_o_ or pH_i_ (Fig 4). This parameter was chosen because it is clearly defined, it does not require forcing data to fit a Boltzmann distribution, and it does not require estimating the threshold of activation, which has been used for this purpose previously by us and others, but is imprecise and arbitrary. These considerations are discussed at greater length elsewhere (see Methods of [37]). When pH_o_ or pH_i_ was varied, the *g*_H_-*V* relationship shifted by ~40 mV/unit change in pH, matching the reference line showing this slope (Fig 4). For both pH_o_ and pH_i_ the shift tended to saturate above pH 8, similar to H_V_1 from rat [19], human, the dinoflagellate *Karlodinium veneficum*, and the coccolithophore *Emiliania huxleyi* [37]. The saturation occurs above typical environmental pH, and likely reflects the approach of pH to the p*K*_a_ of one or more pH sensing sites.

#### LpH_V_1 channel opening kinetics

**Fig 5:**
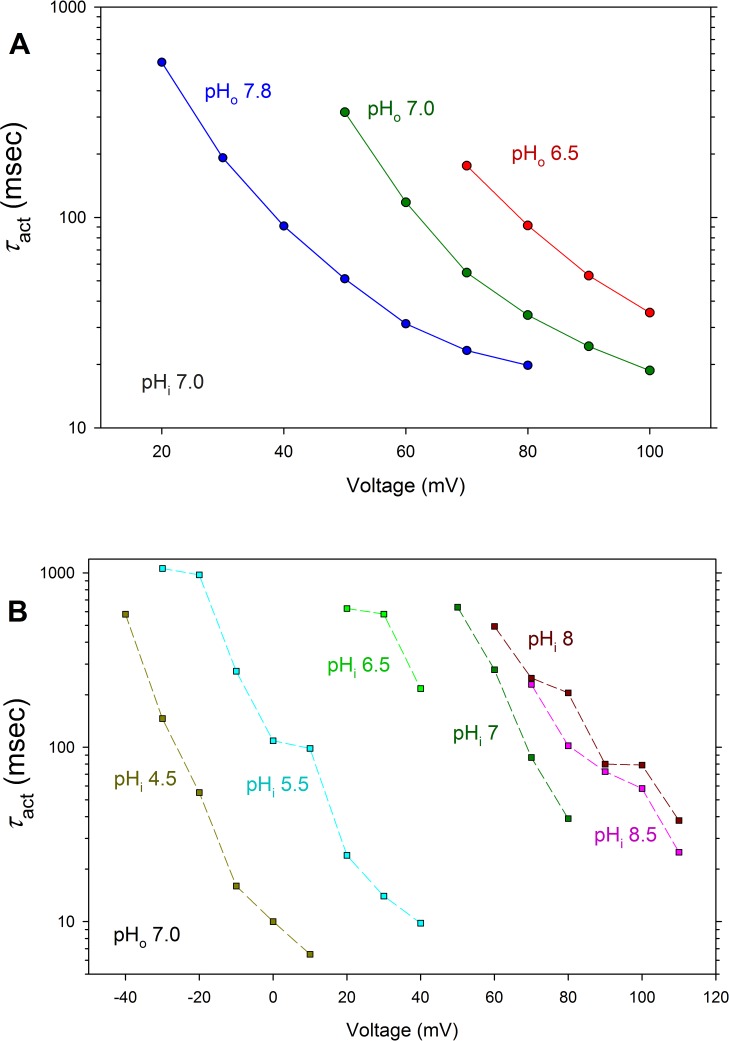
Voltage and pH dependence of LpH_V_1 activation kinetics. **A. Voltage and pH**_**o**_ **dependence of LpH**_**V**_**1 activation kinetics.** Currents were fitted by single rising exponentials to obtain the time constant of channel opening (activation, *τ*_act_). These measurements were made in the same cell with pH_i_ 7.0, studied at three different pH_o_. **B. Voltage and pH**_**i**_ **dependence of LpH**_**V**_**1 activation kinetics.** These measurements were made in the same inside-out patch of membrane with pH_o_ 7.0, studied at six different pH_i_.

The rising current during a depolarizing pulse was fitted with a single exponential function to determine the time constant of activation, *τ*_act_. At symmetrical pH 8 (pH_o_ = pH_i_ = 8), *τ*_act_ ranged 45–600 msec at +60 mV (*n* = 6); at symmetrical pH 7, *τ*_act_ ranged 30–470 msec at +80 mV (*n* = 8). Examples of the dependence of *τ*_act_ on pH are shown for whole-cell measurements (Fig 5A) in which pH_o_ was varied, and for inside-out patches of membrane (Fig 5B) in which pH_i_ was varied. In both configurations, changes in pH appeared to simply shift the *τ*_act_ -*V* relationship along the voltage axis. As in all species, current activation (turn-on) became faster with greater depolarization (Fig 1A–1C). However, in LpH_V_1 this property was markedly exaggerated. Activation became much faster (smaller *τ*_act_) with larger depolarizing pulses; typically, *τ*_act_ was 100 times faster at voltages 60 mV more positive.

### LpH_V_1 stains multiple membranes in intact cells

**Fig 6:**
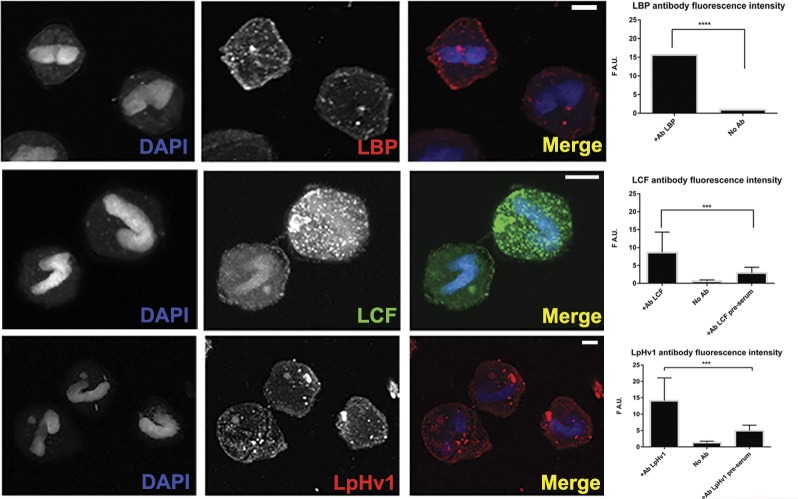
LpH_V_1 distribution is consistent with scintillon localization. Fixed whole *L*. *polyedrum* were probed with antibodies to LBP, LCF, and LpH_V_1, stained with fluorescently labeled secondary antibodies to appropriate IgG, and visualized via confocal microscopy. Maximum projection of a representative Z-stack for each primary antibody is shown. Scale bars in all panels = 10 μM. Images were analyzed for per area fluorescence from each secondary antibody using Zen software tools. Bars represent means +/- S.D. of fluorescence from 20–30 individual cells from 2–5 separate preparations; significant differences from no-antibody or pre-serum controls are indicated with asterisks. Images of negative controls are presented in S1 Fig.

If LpH_V_1 functions to allow protons across the vacuole into the scintillons as predicted, it should be localized in scintillon membranes. The other bioluminescence proteins LCF and LBP were previously demonstrated to localize to scintillons in *L*. *polyedrum* [24,27,30]. We immunostained PFA-fixed, methanol-dehydrated *L*. *polyedrum* cells with chicken anti-LCF, rabbit anti-LBP, or rabbit anti-LpH_V_1. We visualized their localization with organism-specific secondary antibodies, each labeled with a different fluorophore. Western blotting of *L*. *polyedrum* whole cell lysates and purified recombinant LCF, LBP, and LpH_V_1 probed with these primary antibodies detected proteins of the expected sizes and no cross-reactivity (data not shown). The confocal microscopy images in Fig 6 demonstrate that LCF and LBP are distributed in a punctate pattern as previously observed in dark-harvested *L*. *polyedrum* [25]. Fluorescence intensities of labeled cells were significantly different than both pre-serum and no-primary-antibody controls (see also S1 Fig for negative control images). As expected from previous studies [30,38], total fluorescence from antibody-labeled LCF and LBP decreases significantly in cells fixed at mid-light phase compared to mid-dark phase (data not shown).

The proposed function of H_V_1 in bioluminescence requires localization in scintillon membranes (evaginations of the vacuole membrane); nonetheless, no cellular membrane could be excluded *a priori* as a potential site for H_V_1. As seen in Fig 6, a significant fraction of the LpH_V_1 localizes around the periphery of the organism, consistent with an additional plasma membrane location.

### LpH_V_1 co-localizes with LCF and LBP in isolated scintillons

**Fig 7:**
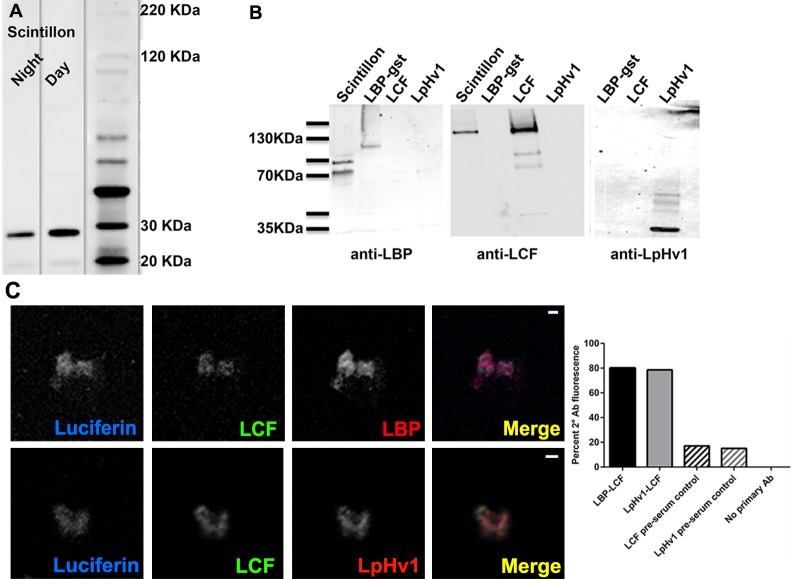
LpH_V_1 localizes to the scintillon. **(A)** Total protein from isolated *L*. *polyedrum* scintillons were Western blotted and probed with anti-LpH_V_1. (**B)** Total protein from isolated *L*. *polyedrum* scintillons, and also purified recombinant LCF, LpH_V_1, and GST-labeled LBP, were Western blotted and probed with the antibody indicated. (**C)** Isolated scintillons were fixed and immunostained as in Fig 6. Scale bars in all panels = 2 μm. Scintillons in different treatments were identified by their native luciferin fluorescence; the percentage of scintillons in each treatment that exhibited secondary antibody fluorescence is shown. Number of scintillons scored for each treatment: LBP-LCF, n = 55; LpH_V_1-LCF n = 55; LCF preserum, n = 41; LpH_V_1 pre-serum, n = 15; no primary antibody, n = 35. Images of negative controls are presented in S2 Fig.

To further explore the cellular localization of H_V_1, we isolated scintillons from *L*. *polyedrum* using density gradient centrifugation [30,39]. Scintillon isolation was confirmed initially by a bioluminescence activity assay [30]. Western blots of proteins extracted from isolated scintillons and probed with antibodies to LBP and LCF demonstrate the presence of these known scintillon markers and also show no detectable cross-reactivity of these antibodies with recombinant LpHv1 protein (Fig 7B). As has been described previously, presumed proteolysis products for LBP and LCF are visible in these Western blots. As seen in Fig 7A, Western blotting of proteins extracted from isolated scintillon preparations shows that the antibody to LpH_V_1 detects a scintillon protein with an apparent size of about 30 kDa; this antibody detects purified recombinant LpH_V_1 at the expected size of 37 kDa. Many possibilities, including different post-translational processing in bacteria compared to dinoflagellates, could explain the apparent size difference between recombinant and native LpH_V_1. Both in purified recombinant protein preparations and in preparations of scintillon protein, our antibody to LpH_V_1 frequently detects protein bands at ~60 and at ~80 kDa, which are consistent with the size of a truncated (60 kDa) or full length (80 kDa) LpH_V_1 dimer. LpH_V_1 has a strongly predicted coiled-coil region in its C-terminus, so it likely dimerizes like H_V_1 from several other species [40–43]. Our antibodies to scintillon proteins do not cross react (Fig 7B). Western blotting from separate preparations consistently shows more LpH_V_1 (~2 fold) in scintillons isolated during the day phase than the night phase (as in Fig 7A), although the difference is not statistically significant.

Scintillon isolation was further confirmed by confocal microscopy of fixed scintillon preparations, using native luciferin fluorescence [44] to positively identify scintillons [25] (Fig 7C). As previously described [25], we observed a low level of contaminating chlorophyll fluorescence. On the slide, chlorophyll fluorescence rarely overlapped with luciferin fluorescence, and many structures with luciferin fluorescence but no nearby chlorophyll fluorescence were visible. Immunostaining of fixed scintillon preparations confirms the presence of LBP and LCF in isolated scintillons (Fig 7C). Immunostaining of fixed scintillon preparations show that LpH_V_1 localizes to luciferin-containing structures that also contain LCF (Fig 7C). That both luciferin and LCF are known markers of the scintillon structure is strong evidence that LpH_V_1 localizes to scintillon membranes.

### Mass spectrometry detects LpH_V_1 peptides in isolated scintillon preparations

**Fig 8:**
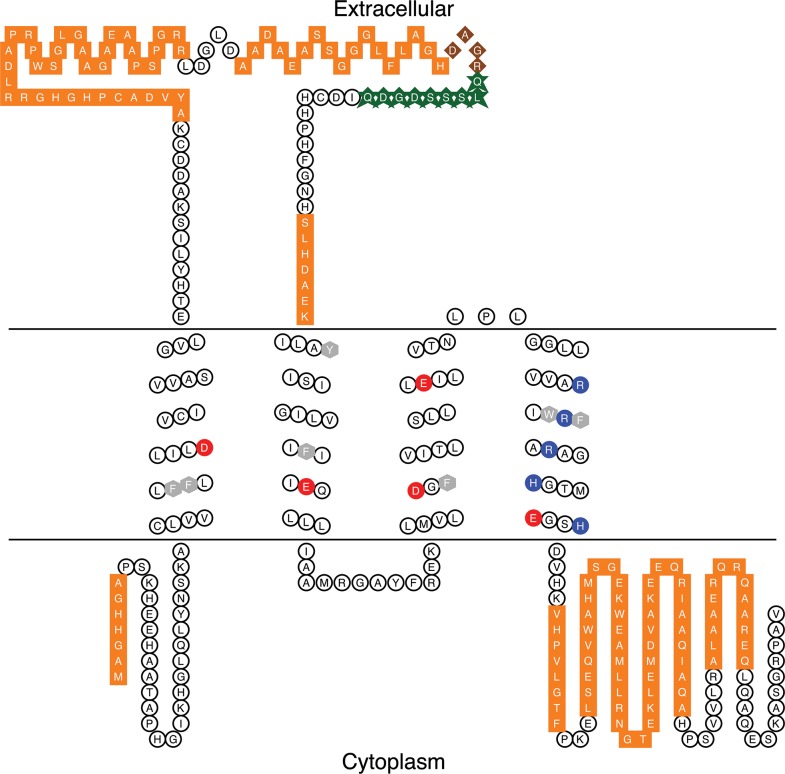
Isolated scintillon preparations contain peptides matching the LpH_V_1 sequence. The sequence and predicted secondary structure acid of LpH_V_1 is shown (Johns S.J., TOPO2, Transmembrane protein display software, http://www.sacs.ucsf.edu/TOPO2/). Acidic residues in the transmembrane helices are shown in red, basic residues in dark blue, and aromatic residues in gray. Brown diamonds indicate the overlap of peptide sequences found by mass spectrometry analysis of isolated scintillons and the epitope to which the antibody against LpH_V_1 was raised; otherwise peptide sequences are shown in orange squares and the epitope is shown in green stars.

To further confirm the presence of LpH_V_1 in scintillons, we subjected proteins from scintillon preparations to tandem mass spectrometry (MS/MS) analysis. We excised five prominent bands from an Imperial stained gel: one at 33 kDa, close to the size expected for LpH_V_1, and others ranging from ~25 to ~50 kDa. We detected 17 different peptides from the 33 kDa band, and 2 different peptides from the 50 kDa band, each with more than one independent peptide spectrum, that met the probability threshold for matching the predicted sequence of LpH_V_1. The sequences of these peptides are mapped onto the predicted sequence of LpH_V_1 in Fig 8. Many distinct peptides from the excised bands met the probability threshold for scintillon marker proteins LBP and LCF. In addition, a large number of peptides with significant matches to known chloroplast proteins (*e*.*g*., ribulose-bisphosphate carboxylase, glyceraldehyde-3-phosphate dehydrogenase, peridinin-chlorophyll a-binding protein, etc.) were detected, consistent with the known chloroplast contamination of the scintillon preparation.

### Zn^2+^ inhibits LpH_V_1 proton currents and the flash induced by acid in isolated scintillons

**Fig 9:**
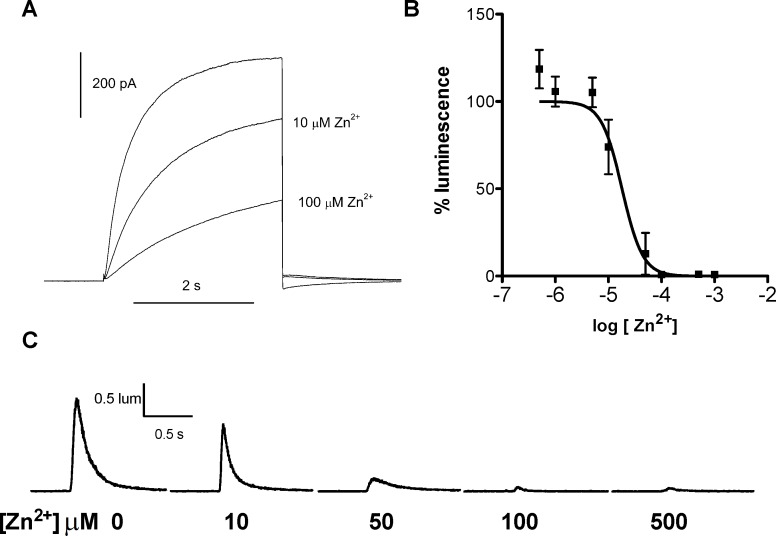
Zn^2+^ inhibits LpH_V_1 proton currents and scintillon luminescence. **(A)** Proton currents at +60 mV at pH_o_ 7.0 were reduced by Zn^2+^. The mean reduction of current by 100 μM Zn^2+^ was 63 ± 11% (mean ± SEM, *n* = 4). (B) Luminescence of *L*. *polyedrum* scintillons stimulated by 50 mM acetate and measured in a plate reader was inhibited by Zn^2+^. (C) Zn^2+^ sensitivity of luminescence of *L*. *polyedrum* scintillons stimulated by 50 mM acetate and measured with a photometer generously provided by J. W. Hastings.

Few H_V_1 inhibitors have been identified; the most potent in many species is Zn^2+^ [45,46]. Fig 9 shows that Zn^2+^ inhibited LpH_V_1 currents detectably at 10 μM, and substantially at 100 μM. At similar concentrations, Zn^2+^ inhibited luminescence elicited by acid exposure in scintillons isolated from *L*. *polyedrum* (Fig 9B and 9C). No flash was elicited when detergent was present, suggesting that intact scintillons were required for the response. These results strongly support the hypothesis that LpH_V_1 is the proton channel that triggers the bioluminescent flash in *L*. *polyedrum*.

## Discussion

In 1972, J. Woodland “Woody” Hastings predicted the existence of voltage-activated proton-selective channels that trigger the bioluminescent flash in *Gonyaulax polyedra* (now *Lingulodinium polyedrum*) [1]. An action potential in the vacuole membrane was known to initiate the flash [34]. The scintillon contains luciferase, its substrate luciferin, and luciferin-binding protein. A drop in pH both activates luciferase [33] and releases luciferin from its binding protein [47]. Because the vacuole pH was 3.5–4.5 [28,48], a proton-permeable channel that was opened by depolarization during the action potential would allow rapid proton flux into the scintillon, releasing luciferin, activating luciferase, and triggering the flash [1]. The present results strongly support this hypothesis.

Luciferin, LBP, and LCF from several species cross react [26,49] supporting a common role of pH, and presumably H_V_1, in the signal transduction pathway. We used RNA-seq data for *L*. *polyedrum* to identify a putative H_V_1 gene. We confirmed that the organism expresses the RNA (S1 Table) and protein (Fig 7) predicted from this gene.

### Structural comparison of LpH_V_1 with other proton channels

LpH_V_1 is the tenth H_V_1 gene to be identified and confirmed by voltage-clamp studies in a heterologous expression system. Globally, LpH_V_1 resembles all other H_V_1 in having four transmembrane helical segments. Compared with hH_V_1, it has a short N terminus, ~33 amino acids vs. ~100 in hH_V_1. LpH_V_1 has a very long S1-S2 linker of ~100 residues vs. ~10 in hH_V_1. S1-S2 linkers over 70 residues long appear in two electrophysiologically confirmed H_V_1 from coccolithophores, and five of the twelve high confidence dinoflagellate H_V_1 shown in S3 Fig. These long linkers do not share significant similarity to each other, and BLAST searches of the linker region reveal little similarity to proteins of known function. A region of about 20 amino acids (351–373) in the LpH_V_1 linker is predicted to be helical by several secondary structure prediction programs. LpH_V_1 most likely exists as a dimer, because it has a strongly predicted coiled-coil region in its C-terminus. H_V_1 from several other species are thought to dimerize, in large part enforced by C terminal coiled-coil interactions [40–43,50].

The sequence of LpH_V_1 includes the amino acids thus far identified to play critical roles in the function of the molecule in other species [51]. LpH_V_1 shares the “signature sequence” of all other H_V_1 that includes an Asp in S1 and the motif RxWRxxR in S4 [12,14]. The Asp in the middle of the S1 segment is crucial to establishing proton selectivity [9,12,14,16], the Arg in S4 are thought to confer voltage sensing as in other VSDs, and the Trp affects multiple properties of H_V_1 [37]. LpH_V_1 has residues (Phe^171^, Leu^42^ and Ile^199^) that we propose form the hydrophobic gasket that has been identified in other voltage gated ion channels [52–55]. These three hydrophobic residues are aligned horizontally near the middle of the membrane, where they separate internally and externally accessible aqueous vestibules. The position occupied by Phe^171^, identified as a delimiter of the charge transfer center [56], is conserved almost universally among VSD-containing molecules.

A number of acidic amino acids are thought to stabilize the channel in closed, open, and intermediate states by electrostatic interactions with the cationic charges in S4. Likely countercharges in the S1 helix in hH_V_1 include Asp^112^, Glu^119^, Asp^123^, and possibly Lys^125^ [51]. The corresponding positions in LpH_V_1 are Asp^45^, Ser^52^, Glu^56^, and His^58^. When externally-accessible acidic groups are neutralized by mutation, the *g*_H_-*V* relationship shifts positively, because the open state is disfavored [51]. The somewhat more positive activation of LpH_V_1 may reflect the absence of Glu^119^ and Lys^125^. On the other hand, the other identified dinoflagellate H_V_1, kH_V_1 activates 60 mV more negatively than all other species, despite kH_V_1 lacking Glu^119^ (with Gly instead). The important countercharges in S2 and S3, are conserved in all identified H_V_1 including LpH_V_1, with its Glu^174^ and Asp^195^. Another acid in S3 unique to H_V_1 is Asp^185^ (hHv1 numbering), which is absent in other VSDs [12,51]. In LpH_V_1 this position is occupied by the conservative substitution Glu^206^. In summary, with the exception of one acidic residue in S1, the charges in the transmembrane region of LpH_V_1 are quite similar to those in other H_V_1.

### The electrophysiological properties of LpH_V_1 are consistent with its proposed function in triggering the flash

We measured several biophysical properties of the LpH_V_1 channel to determine whether the LpH_V_1 gene product has properties consistent with its proposed function of triggering the bioluminescent flash. These properties include 1) proton selectivity, 2) activation by depolarization, 3) opening kinetics comparable to that of the flash, and 4) the ability to conduct inward current (from vacuole to cytoplasm), all of which should occur *in vivo*. Our experiments were done at room temperature (20–25°C) which is within the range of oceanic temperatures, but other possible differences between our experimental conditions and those *in vivo* cannot all be evaluated so easily. The mammalian cells used as an expression system (HEK-293 cells) may process proteins differently than dinoflagellates and likely have a different membrane composition than *L*. *polyedrum*. Salt concentrations appropriate for mammalian cells, ~300 mOsm, are about half those of sea water. A critical factor for the H_V_1 protein is the pH gradient. The cytoplasmic pH in dinoflagellates is estimated to be pH 8.0 [1], but the vacuolar pH in bioluminescent species *L*. *polyedrum* [28] and *Noctiluca* [48] is 4.5 and 3.5, respectively. HEK-293 cells did not survive such a large pH gradient. The membranes of mammalian cells tolerate neither extreme pH *per se*, nor extreme pH gradients. Our experiments address the posed questions with these constraints.

LpH_V_1 meets the first two requirements: it is clearly highly proton selective (Fig 2) and it opens with depolarization (Fig 1). The next question is whether the kinetics of LpH_V_1 match that of the bioluminescent flash. The flash recorded from individual *L*. *polyedrum* had a latency of 15–22 msec [57] and the flash in individual scintillons isolated from *L*. *polyedrum* or from entire organisms had a time-to-peak of ~100–200 msec [25,58]. LpH_V_1 opens faster than H_V_1 of mammalian species (where *τ*_act_ is measured in seconds), but slower than that of snail neurones with *τ*_act_ of a few milliseconds [18]. As in all species, LpH_V_1 channels open faster with increasing depolarization. Differing sharply from other species, the activation kinetics of LpH_V_1 depends quite steeply on voltage. As evident in Fig 5, *τ*_act_ was ~1 s just above *V*_threshold_ but became ~100 times faster within 50–60 mV, changing *e*-fold in just ~10 mV. In contrast, in several mammalian or amphibian cells, the voltage required to change *τ*_act_ *e*-fold ranges 40–80 mV [3,59–63]. As a result of the steep voltage dependence of *τ*_act_, a large depolarization could activate LpH_V_1 within ~10 msec. The kinetics of LpH_V_1 thus seems consistent with the kinetics of the bioluminescent response. Density of LpH_V_1 expression in the native membrane and the presence of other ion channels would also modulate the response.

We also addressed whether LpH_V_1 could produce inward H^+^ current during an action potential *in situ*. At symmetrical pH (e.g., pH_o_ 7, pH_i_ 7) inward current was not observed. With an inward pH gradient of 1–2 units (e.g., pH_o_ 7, pH_i_ 9), however, inward current was observed. An example is shown in Fig 1D. Once inward H^+^ current is activated in the tonoplast, the resulting H^+^ influx would further depolarize the membrane regeneratively, opening more channels and driving the membrane potential toward *E*_H_, which for pH 4.5//8.0 is in the vicinity of +200 mV. Correspondingly, action potential peaks of 200 mV have been recorded *in situ* in *Noctiluca* [21]. We were at first surprised that LpH_V_1 activated relatively positively, compared with other H_V_1 [20] and especially when compared with kH_V_1 that activates well negative to *E*_H_ [12]. Perhaps, given the enormous inward H^+^ gradient across the *L*. *polyedrum* tonoplast, it would be perilous for the cell to allow LpH_V_1 activation except when triggering a flash. *K*. *veneficum* is not bioluminescent, and the function of kH_V_1 is uncertain, but is likely different from that in bioluminescent species.

### The cellular localization of LpH_V_1 is consistent with its proposed role in bioluminescence

Three lines of evidence demonstrate convincingly that LpH_V_1 is expressed in the vacuole membrane surrounding the scintillons, confirming its predicted role in bioluminescence: 1) Western blots of isolated scintillons show antibody staining of a protein with the predicted size and no detectable cross-reactivity with LCF or LBP (Fig 7); 2) immunostaining of isolated scintillons with the same antibodies shows that LpH_V_1 colocalizes with LCF, a marker of the scintillon organelles (Fig 7); and 3) proteomics analysis of proteins extracted from isolated scintillons shows the expected presence of LBP and LCF proteins (S2 Table) and the presence of LpH_V_1 protein (Fig 8) in these structures. Further confirmation that LpH_V_1 is present in scintillons is the inhibition of the flash in isolated scintillons by Zn^2+^ at concentrations that inhibit LpH_V_1 proton currents (Fig 9).

Upon stimulation by shear stress, mechanosensor (probably stretch activated) channels at the surface of *L*. *polyedrum* [58,64] are thought to relay a signal through intracellular calcium signaling via G-proteins [65,66], but the molecular identities of the signaling components that result in the action potential at the vacuole membrane and subsequent luminescence are unknown. It is likely that LpH_V_1 mediates the action potential in the vacuole membrane that triggers the flash, because H^+^ is the only ion with a sufficiently positive Nernst potential to generate an action potential that peaks at +200 mV [21]. Voltage gated Na^+^ channels have been reported in the outer membrane of *Noctiluca*, but their reversal potential in sea water is only +33 mV [67]. Although Hastings’ original proposal required a proton channel to open only during the action potential in scintillon membranes, it was later realized that the same channel could also mediate the action potential in the tonoplast [28,68]. We have identified additional putative H_V_1 sequences in RNA-seq data from two other bioluminescent dinoflagellates, *Alexandrium tamarense* and *Noctiluca scintillans*, excellent evidence that H_V_1, like LCF and LBP, is a conserved component of the signal transduction pathway that leads from shear stress at the organism surface to the light flash.

Immunostaining of whole cells suggests that membranes other than those surrounding the scintillons may contain LpH_V_1 (Fig 6). The non-bioluminescent dinoflagellate *K*. *veneficum* expresses a H_V_1 in feeding populations at night [12]; we have identified putative H_V_1 genes in several additional dinoflagellates (S3 Fig) based on the presence of an Asp in the middle of S1 crucial to proton selectivity [9,12,14] and a signature sequence in S4 involved in gating (10). In other organisms H_V_1 functions in many processes [20], raising the likelihood that in *L*. *polyedrum* LpH_V_1 also serves purposes in addition to the control of bioluminescence. The dinoflagellates (bioluminescent or not) in which we found H_V_1 gene sequences span a large fraction of the dinoflagellate phylogenetic tree [69,70]. Taken together, these data suggest that primordial H_V_1 functions have been co-opted by the bioluminescent species for light production. Intriguingly, antibody to LCF stained trichocysts in *L*. *polyedrum* [28], a tantalizing hint of possible non-bioluminescent functions for the bioluminescence enzyme.

## Materials and methods

### Sequence searching and alignments

The *K*. *veneficum* voltage-gated proton channel (kH_V_1, NCBI accession JN255155) was used as a BLAST probe with e-value cutoff of 10^−2^ against the Marine Microbial Eukaryote Transcriptome Sequence Project (MMETSP; [36]) which yielded a single contig (26874) in MMETSP1032. BLAST searches of the non-redundant database at NCBI using the translation of this contig yielded H_V_1 from diatoms and coccolithophores with e-values of 10^−11^. Using various H_V_1 probes, searches of MMETSP also yielded full and partial sequences for putative H_V_1s from *Akashiwa sanguinea*, *Alexandrium monilatum*, *Alexandrium tamarense*, *Amphidinium carterae*, *Azadinium spinosum*, *Karenia brevis*, *Noctiluca scintillans*, *Symbiodinium*, *and Scrippsiella trocoida*. We included a recently described putative H_V_1 from *Prorocentrum minimum* [71]. These sequences were aligned with a set of high confidence animal H_V_1s using MSAprobs [72] (S3 Fig). The predicted secondary structure for LpH_V_1 was drawn using TOPO2 (Johns S.J., TOPO2, Transmembrane protein display software, http://www.sacs.ucsf.edu/TOPO2/).

### Gene and antibody synthesis

Genes for LpH_V_1 (based on longest open reading frame of RNA-seq contig 19215 from the *L*. *polyedrum* library) and LCF (NCBI accession AF085332.1), codon-optimized for mammalian (LpH_V_1) or *E*. *coli* (LCF) expression, were synthesized by Genscript Corp. and subcloned into pcDNA and pEGFP (LpHv1) or pQE-30 (LCF). Peptides corresponding to chosen epitopes from LpH_V_1 (CDAGRQLSSDGDQ) and LCF (CLDYPKKRDGWLEKN) were synthesized by Genscript and polyclonal antibodies to these peptides were raised and affinity purified from rabbit (LpH_V_1, final protein concentration 1.1 mg/ml) or chicken (LCF, final protein concentration 0.88 mg/ml). Preserum from animals was also provided by Genscript. The native gene for LBP in pGEX-4T, and rabbit antibody raised to LBP, were the generous gifts of Dr. David Morse (University of Montreal).

### Electrophysiology

HEK-293 cells were grown to ~80% confluence in 35 mm cultures dishes. Cells were transfected with 0.4–0.5 μg of cDNA using Lipofectamine 2000 (Invitrogen) or PEI (polyethylenimine, Sigma). After 6 h at 37°C in 5% CO_2_, cells were trypsinized and re-plated onto glass cover slips at low density for patch clamp recording the following day. We selected green cells under fluorescence for recording. Whole-cell or excised inside-out patch configurations of the patch-clamp technique were carried out as described in detail previously [73]. Bath and pipette solutions were used interchangeably. They contained (in mM) 2 MgCl_2_, 1 EGTA, 80–100 buffer, 75–120 TMA^+^ CH_3_SO_3_^–^ (to bring the osmolality to ~300 mOsm), titrated using TMAOH. Buffers with p*K*_a_ near the desired pH were used: Homopipes for pH 4.5–5.0, MES for pH 5.5–6.0, BisTris for pH 6.5, BES for pH 7.0, HEPES for pH 7.5, Tricine for pH 8.0, CHES for pH 9.0, and CAPS for pH 10. Experiments were done at 21°C or at room temperature (20–25°C). Current records are shown without leak correction.

Reversal potentials (*V*_rev_) in most cases were determined from the direction and amplitude of tail current relaxation over a range of voltages, following a prepulse that activated the proton conductance, *g*_H_. When the *g*_H_ activated negative to *V*_rev_ the latter could be determined directly from families of currents. Currents were fitted with a single exponential to obtain the activation time constant (*τ*_act_) and the fitted curve was extrapolated to infinite time to obtain the “steady-state” current amplitude, from which the *g*_H_ was calculated. The voltage at which *g*_H_ was 10% of *g*_H,max_ (*V*_*g*H,max/10_) was determined after defining *g*_H,max_ as the largest *g*_H_ measured.

### Cell culture

*L*. *polyedrum* (CCMP 1932, obtained from National Center for Marine Algae and Microbiota-Bigelow Laboratory for Ocean Sciences) cultures were grown in three locations with minor differences in conditions. Cultures were grown in L1 minus Si [74] or F/2 minus Si [75] medium prepared in artificial seawater (Instant Ocean, Blacksburg) and maintained in 12:12 or 14:10 light:dark cycle (photon flux 100 μmoles/m^2^/s) at 18–20°C. Cultures were allowed to grow until reaching a cell density of 4,000–10,000 cells/ml at which point they were collected at mid-dark and mid-day time points.

### RNA extraction, qPCR analysis, and cDNA cloning

Primer sets covering the entire predicted LpHv1 coding sequence (S1 Table) were used for qPCR analysis performed on cells harvested at mid-light or mid-dark. Cells from 50 ml aliquots of culture were collected by centrifugation. The pellet was used immediately or flash frozen in an ethanol and dry ice bath and stored at -80°C. Samples from each timepoint were resuspended and Dounce homogenized in 1 ml TRI reagent (Zymoresearch), and RNA was extracted according to the manufacturer's protocol. RNA was reverse transcribed using Superscript II reverse transcriptase (Invitrogen by Life Technologies) with random primers (Invitrogen) according to the manufacturer's protocol. Quantitative real-time PCR was performed in triplicate using an Applied Biosystems (Life Technologies) Fast 7500 thermal cycler with primers listed in S1 Table. Thermal cycling conditions consisted of an initial denaturation at 95°C for 2 minutes followed by 40 cycles of denaturation at 95°C for 15 seconds, annealing and fluorescent data collection at 60°C for 15 seconds, and extension at 72°C for 30 seconds. Cycle thresholds and baselines were determined manually and cycle thresholds were averaged and compared across time points.

### Recombinant protein expression and purification

Recombinant LBP and LpH_V_1 proteins tagged with glutathione s-transferase (GST) were expressed in *E*. *coli* by induction with isopropyl b-d-1 thiopyranogalactoside (IPTG) at 23°C (LBP) or 17°C (LpH_V_1) for 8–24 hours. Recombinant LCF protein tagged with 6-His was expressed in *E*. *coli* by induction with IPTG at 17 C for 16–24 hours. Proteins were purified from *E*. *coli* according to the manufacturer’s instructions (GE-Healthcare for LBP and LpH_V_1, Qiagen for LCF). GST tag was cleaved from LpH_V_1 by digestion with Prescission protease according to the instructions of the vendor. In one experiment, GST tag was cleaved from LBP by digestion with thrombin for 16 hours; the insoluble LBP precipitate was pelleted by centrifugation and solubilized in SDS-PAGE loading buffer.

### Scintillon isolation, luminescence assays, and gel analysis

Scintillons were isolated from cultures grown in two different locations and isolated using a sucrose [39] or a Percoll [30] density gradient. For luminescence assays to test metal sensitivity, the 0.5 ml fraction from a sucrose gradient with the highest luminescence was kept separate and considered to comprise “pure scintillons”. This fraction was pelleted, washed with buffer, resuspended and then diluted to about 1 ml in the extraction buffer. 100 μl of the diluted scintillon preparation was added to wells of a 96 well plate. 50 μl of 5x metal ion/drug solution was added to the same well. The reaction was started by injecting 150 μl of 0.05 M acetic acid and luminescence was measured immediately using pClamp software (Molecular Devices). No flash was detected in the presence of detergents (0.03–0.1% TWEEN-20 or 0.1% SDS).

For gel analysis, total scintillon protein was extracted by heating for 10 min at 95°C in SDS-PAGE sample buffer. Total scintillon proteins, and preparations of purified recombinant proteins, were separated by SDS-PAGE, Western blotted onto PVDF, blocked with 5% milk or Licor blocking buffer (Licor) and probed with diluted antibodies to LCF (1:1000), LBP (1:10000), and LpH_V_1 (1:3000). Blots were visualized with secondary antibodies to appropriate animal IgG conjugated either to HRP or to Licor fluorescent tags. Western blotting results from different scintillon sources were directly comparable.

### Fixation and immunostaining of cells and isolated scintillons

We used the procedure of [76] with modifications. Preliminary experiments indicated that low speed centrifugation was optimum to preserve cell integrity so all centrifugation steps were performed at 300 x g. Cells were pelleted from 50–100 ml of culture by centrifugation, washed with seawater, and fixed in 4% Paraformaldehyde in seawater at room temperature. Fixed cells were washed with phosphate buffered saline (PBS), permeabilized in 100% methanol at 4°C, washed at room temperature with PBS supplemented with 0.1% tween and 1% BSA (PBST-BSA), then blocked by incubation in PBST-BSA at room temperature. Primary antibodies, or serum controls diluted to the same protein concentrations, or vehicle controls, were added and incubated overnight at 4°C. Cells were collected by centrifugation and washed at room temperature with PBST-BSA. For detection, Alexa555-Fluor-conjugated goat anti-rabbit IgG (H+L) and/or Alexa555-Fluor-conjugated goat anti-chicken IgG (ThermoFisher, Waltham MA) was added and incubated in the dark at room temperature. Cells were washed with PBST-BSA at room temperature, incubated with 4',6-diamidino-2-phenylindole (DAPI) 5μg/mL in PBS, and washed again. Cells were incubated in 2 drops of Vectashield overnight at 4°C in the dark and 15 to 50 μL were mounted on slides. Fixation and immunostaining of isolated scintillons were performed as described, but without the methanol permeabilization step. Cells and scintillons were visualized using a Zeiss LSM 700 confocal microscope, equipped with a 20x (whole cells) and 40x (scintillons) 1.2 NA C-Apochromat objective. For immunofluorescent localization, all channel pinholes were set to 1 Airy Unit. Isolated scintillons identified by luciferin fluorescence were scored for the presence or absence of secondary antibody fluorescence. Confocal slices or maximum intensity projections of the Z-stack were rendered using Zeiss Zen software, and processed using Adobe Photoshop.

### Preparation of scintillon proteins for mass spectrometry

Purified scintillon preparations were concentrated by ultra-centrifugation at 4°C. The pellet was re-suspended in SDS sample buffer, heated for 5 minutes at 95°C, and cleared by centrifugation at 10,000 × g. 50 μl of sample was loaded and run on NovexNuPAGE 4–12% bis-tris gels according to the manufacturer’s protocol. Gel bands corresponding to the location of the presumptive proteins were excised with a clean scalpel. Samples were processed using the Thermo Scientific In-Gel Tryptic Digestion Kit according to manufacturer’s protocol. Gel bands were destained twice with 200 μl destaining solution (~25 mM sodium bicarbonate in 50% acetonitrile) and incubated at 37°C with shaking for 30 minutes. Samples were reduced by incubation at 60°C for 10 min in 50mM TCEP (tris(2-carboxyethyl)phosphine) in 25 mM ammonium bicarbonate buffer. Free sulfhydryl groups were alkylated by incubation in 100 mM iodoacetamide at room temperature for 1 hour in the dark. Gel pieces were shrunk in acetonitrile. For the initial proteomics run samples were treated overnight with 100 ng trypsin at 30°C. The second proteomics run samples were treated with 100ng typsin and digestion was performed at 50°C at high pressure using the PBI Barocyler (Pressure Biocsciences Inc.) according to manufacturer’s protocol. Samples were dried in a SpeedVac.

### Mass spectrometry analysis and data processing

Scintillon samples were analyzed by electrospray ionization on an Elite tandem orbitrap mass spectrometer (Thermo Scientific Inc). Nanoflow HPLC was performed by using a Waters NanoAcquity HPLC system (Waters Corporation). Peptides were trapped on a fused-silica pre-column (100 μm i.d. 365 μm o.d.) packed with 2 cm of 5 μm (200 Å) Magic C18 reverse-phase particles (Michrom Bioresources, Inc). Subsequent peptide separation was conducted on a 75 μm i.d. x 180 mm long analytical column constructed in-house and packed with 5 μm (100 Å) Magic C18 particles, using a Sutter Instruments P-2000 CO_2_ laser puller (Sutter Instrument Company). The mobile phase A was 0.1% formic acid in water and mobile phase B was 0.1% formic acid in acetonitrile. Peptide separation was performed at 250 nL/min in a 95 min run, in which mobile phase B started at 5%, increased to 35% at 60min, 80% at 65min, followed by a 5 min wash at 80% and a 25 min re-equilibration at 5%. Ion source conditions were optimized by using the tuning and calibration solution recommended by the instrument provider. Data were acquired by using Xcalibur (version 2.8, Thermo Scientific Inc.). MS data was collected by top-15 data-dependent acquisition. Full MS scan of range 350–2000 m/z was performed with 60K resolution in the orbitrap followed by collision induced dissociation (CID) fragmentation of precursors in iontrap at normalized collision energy of 35. The MS/MS spectra of product ions were collected in rapid scan mode.

Acquired tandem mass spectra were searched for sequence matches against UniprotKB database using COMET. The following modifications were set as search parameters: peptide mass tolerance at 10 ppm, trypsin digestion cleavage after K or R (except when followed by P), one allowed missed cleavage site, carboxymethylated cysteines (static modification), and oxidized methionines (variable modification/differential search option). PeptideProphet and ProteinProphet, which compute a probability likelihood of each identification being correct, were used for statistical analysis of search results. PeptideProphet probability ≥ 0.9 and ProteinProphet probability ≥ 0.95 were used for positive identification at an error rate of less than 1%. Only proteins identified by more than one unique peptide sequence were included in the study.

## Supporting information

S1 Table Primers and predicted product sizes for qPCR. (DOCX)

S2 Table Assignments of peptides via MS/MS analysis of protein from isolated scintillons of *Lingulodinium polyedrum*. Tabs show assignments of proteins from two independent scintillon preparations. Samples shown in ‘MS results 1’ tab were initially treated with 100 ng trypsin at 30°C; those in ‘MS results 2’ tab were initially treated with 100 ng trypsin at 50°C at high pressure using the PBI Barocyler (Pressure Biocsciences Inc.) according to manufacturer’s protocol.
(XLSX)

S3 Table Sequence of longest open reading frame of contig 26784 in MMETSP1032 assembly, and sequence of codon-optimized gene of LpH_V_1. (DOCX)

S1 Fig Fluorescence images from pre-serum or no-antibody control treatments of fixed whole cells. Cells and images were prepared as for Fig 6.
(TIF)

S2 Fig Fluorescence images from pre-serum or no-antibody control treatments of fixed isolated scintillons. Scintillons and images were prepared as for Fig 7.
(TIF)

S3 Fig Alignment of predicted amino acid sequences of putative dinoflagellate H_V_1s. Sequences were found by BLAST searches of RNA-seq projects [35,36] and were aligned with MSA-Probs [72]. Dinoflagellate sequences are shown aligned with sequence logos of individual transmembrane helices obtained from an alignment of animal H_V_1s [12]. The sequence logos are numbered for hH_V_1. Overlapping partial sequences from *Alexandrium monilatum* are not shown.
(TIF)
