# Supplementary material for: Identification of a vacuolar proton channel that triggers the bioluminescent flash in dinoflagellates
Source: PLoS One. 2017 Feb 8;12(2):e0171594. doi: 10.1371/journal.pone.0171594 (PMC5298346; doi:10.1371/journal.pone.0171594)
Supplement: S1 Table — (DOCX) [file pone.0171594.s001.docx]

|  | SEQUENCE | PRODUCT SIZE | TM (°C) |
| --- | --- | --- | --- |
| HV1_1_F | CTTCAAAGCACGAGGAGCAT | 157 | 60.54 |
| HV1_1_R | AGGTAGTGCGTCTCCAGGAC | 157 | 59.33 |
| HV1_2_F | ACTGCAAGGCCTACGTGGA | 185 | 61.81 |
| HV1_2_R | GTGCCCAGCTAGGAAGAGG | 185 | 59.96 |
| HV1_3_F | GGCATCCTCGTGATCTTCAT | 141 | 60.04 |
| HV1_3_R | CAGGTTCGTCACCAGGATCT | 141 | 60.11 |
| HV1_4_F | AGAAGCTGATGGTGCTGGAC | 200 | 60.42 |
| HV1_4_R | GGAGAGCTCCTTGGGAAAAG | 200 | 60.32 |
| HV1_5_F | CTTCAGGGAGAAGCTGATGG | 196 | 59.94 |
| HV1_5_R | GGGAAAAGTTCCCAGGACA | 196 | 59.89 |
