# Supplementary material for: Identification of a vacuolar proton channel that triggers the bioluminescent flash in dinoflagellates
Source: PLoS One. 2017 Feb 8;12(2):e0171594. doi: 10.1371/journal.pone.0171594 (PMC5298346; doi:10.1371/journal.pone.0171594)
Supplement: S3 Table — (DOCX) [file pone.0171594.s003.docx]

S3. Sequences of LpHv1

LpHv1 sequence from transcriptome

>MMETSP1032_1_(paired)_contig_26874

CCGCGGCCGCGGCCGCCGGCCGATGGCTGGGCATCATGGAGCGCCTTCAAAGCACGAGGAGCATGCCGCAACGGCGCCGCACGGCATCAAGCACGGCCTGCAGCTGTACAACAGCAAGGCCTGCCTGGTGGTCCTGTTCTTCCTGCTGATCCTGGACGTGTGCATCGTCGTGGCATCGGGCGTCCTGGAGACGCACTACCTGATCTCCAAGGCCGACGACTGCAAGGCCTACGTGGACGCCTGCCCCCACGGCCACGGCCGCCGCCTCGACGCCCCGCGCCCGTGGAGCGGCCTCGGCGCCGCCGGCGCAGAAGCCGCGCCCTCGCCCGGGCGCCGACTGGACGGCCTCGACGCCGCTGACGCAGAAGCGAGCAGCGGCGGCGGCCTCTTCCTAGCTGGGCACGACGCGGGGCGGCAGCTGAGCTCTTCGGACGGCGACCAGATCGACTGCCACCACCCGCACTTCGGCAACCACAGCCTGCACGACGCCGAGAAGATCCTGGCCTACATCTCCATCGGCATCCTCGTGATCTTCATCATCGAGCAGCTGCTGCTCATCGCCGCGATGAGGGGCGCGTACTTCAGGGAGAAGCTGATGGTGCTGGACGGCTTCGTGATCACCCTCTCGCTGCTGCTGGAGATCCTGGTGACGAACCTGCCGCTGGGCGGCCTCCTCGTCGTCGCGCGCATCTGGCGCTTCGCCCGGGCGGGGCACGGCACCATGGAGGGCTCGCACGACGTGCACAAGGTGCACCCTGTCCTGGGAACTTTTCCCAAGGAGCTCTCCGAGCAGGTCTGGGCCCACATGTCAGGCGAGAAGTGGGAGGCCATGCTCCTGCGCAACGGCACGGAGAAGCTGGAGATGGACGTGGCAAAGGAGGAGCAGCGCATCGCGGCCCAGATCGCGCAGGCCCACCCCAGCGTCGTGCTCCGCGCCCTGGCCGCGGAGCGCCAGCGCCAGGCCGCCCGCGAGCAGCTGCAGGCGCAGGAGAGCAAGGCCTCGGGCCGGCCGGCCGTTTGAGGCCCGCGCCGCCCCCCCCGCGCCCCTGGAGGGGGCGCACGCCGCCACGCCTT

LpHv1 DNA sequence, optimized for mammalian codons

>LpHv1 optimized for mammalian expression ATGGCAGGACATCACGGAGCACCAAGCAAGCACGAAGAGCACGCCGCAACCGCCCCTCACGGAATCAAGCACGGACTGCAGCTGTATAACAGCAAGGCCTGCCTGGTGGTCCTGTTCTTTCTGCTGATTCTGGATGTCTGTATCGTGGTCGCCTCTGGCGTGCTGGAGACACATTACCTGATTAGTAAGGCTGACGATTGCAAAGCCTATGTGGATGCTTGTCCACACGGACATGGCCGGAGACTGGACGCACCCAGACCTTGGTCAGGACTGGGAGCAGCTGGAGCTGAGGCAGCACCAAGCCCCGGGAGGCGCCTGGACGGACTGGATGCTGCAGACGCAGAAGCTAGCTCCGGAGGAGGACTGTTCCTGGCAGGACATGATGCCGGCAGGCAGCTGTCTAGTTCAGACGGCGATCAGATTGATTGCCACCATCCCCACTTCGGGAACCACTCCCTGCATGACGCTGAAAAGATCCTGGCATACATCTCTATTGGCATCCTGGTCATCTTCATCATTGAGCAGCTGCTGCTGATCGCCGCTATGCGAGGGGCCTATTTCAGGGAAAAACTGATGGTCCTGGACGGATTTGTGATTACCCTGAGTCTGCTGCTGGAGATCCTGGTGACAAATCTGCCTCTGGGAGGACTGCTGGTGGTCGCAAGAATCTGGAGGTTCGCACGAGCAGGACATGGAACTATGGAAGGCTCTCACGATGTGCATAAGGTCCACCCTGTGCTGGGGACCTTTCCAAAAGAGCTGAGCGAACAAGTGTGGGCCCACATGTCCGGAGAGAAATGGGAAGCTATGCTGCTGCGGAATGGCACCGAGAAGCTGGAAATGGACGTGGCCAAAGAGGAACAGCGCATTGCAGCCCAGATCGCTCAGGCACACCCCTCCGTGGTCCTGCGGGCCCTGGCTGCAGAGCGACAGCGGCAGGCCGCAAGAGAACAGCTGCAGGCTCAGGAAAGCAAGGCATCAGGAAGACCCGCCGTGTGA
