# Supplementary figures and images for: Identification of a vacuolar proton channel that triggers the bioluminescent flash in dinoflagellates
Source: PLoS One. 2017 Feb 8;12(2):e0171594. doi: 10.1371/journal.pone.0171594 (PMC5298346; doi:10.1371/journal.pone.0171594)

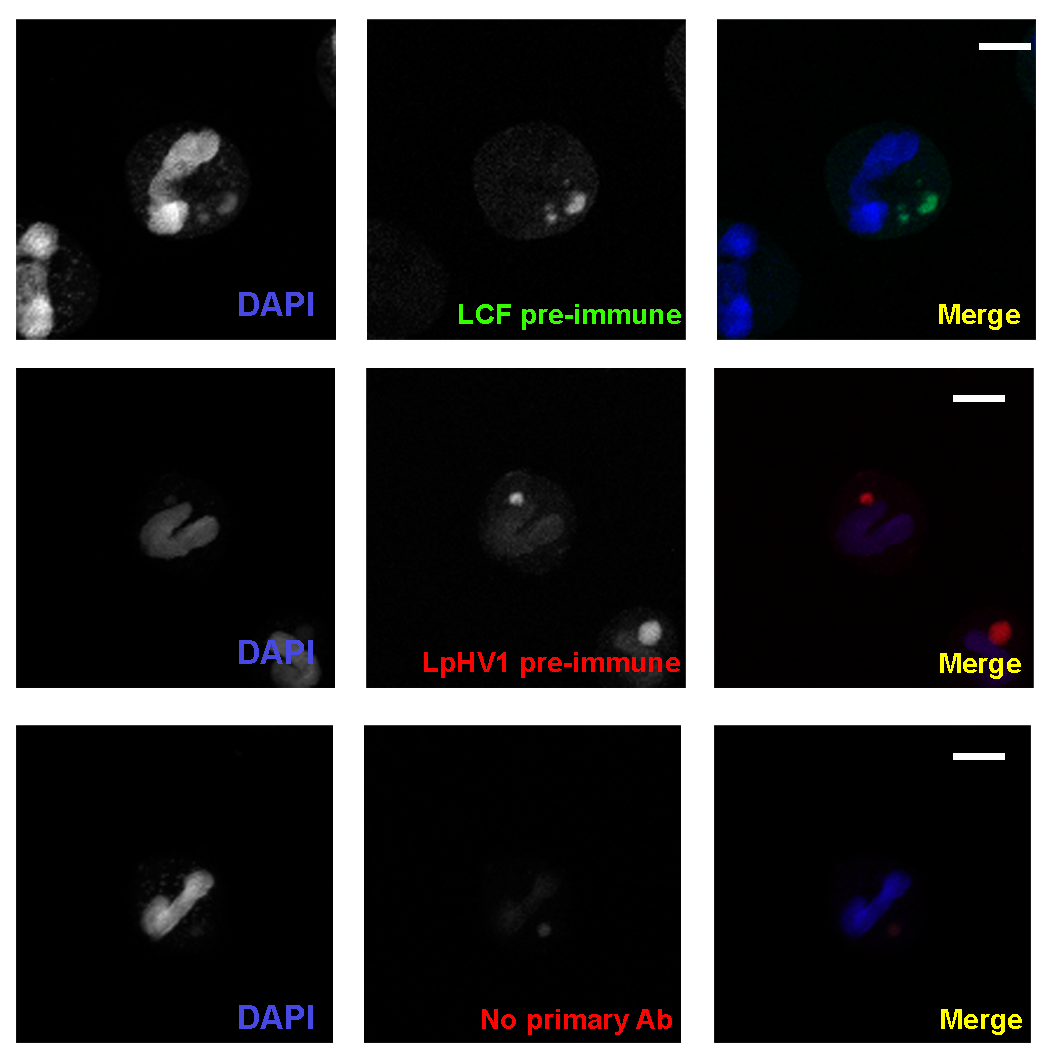

Supplement: S1 Fig — Cells and images were prepared as for Fig 6. (TIF) [file pone.0171594.s004.tif]

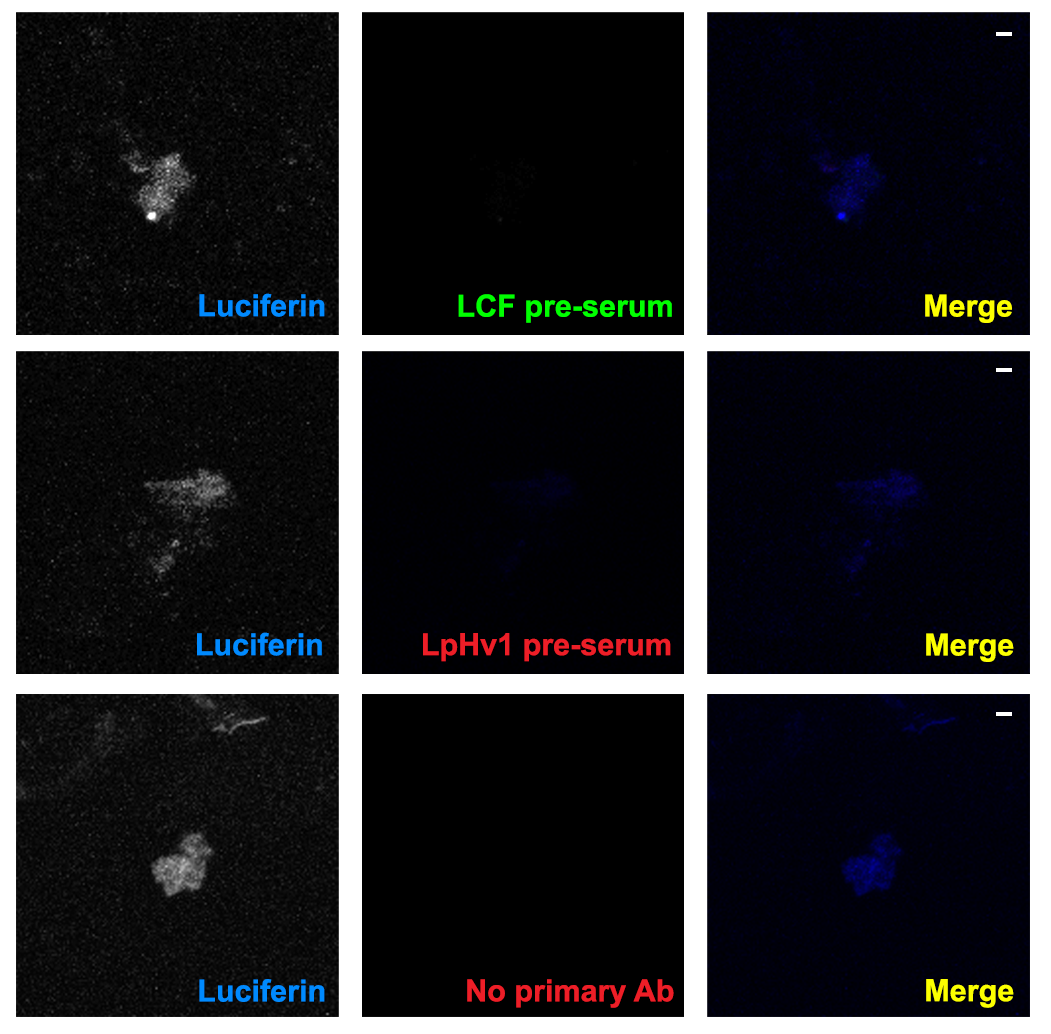

Supplement: S2 Fig — Scintillons and images were prepared as for Fig 7. (TIF) [file pone.0171594.s005.tif]

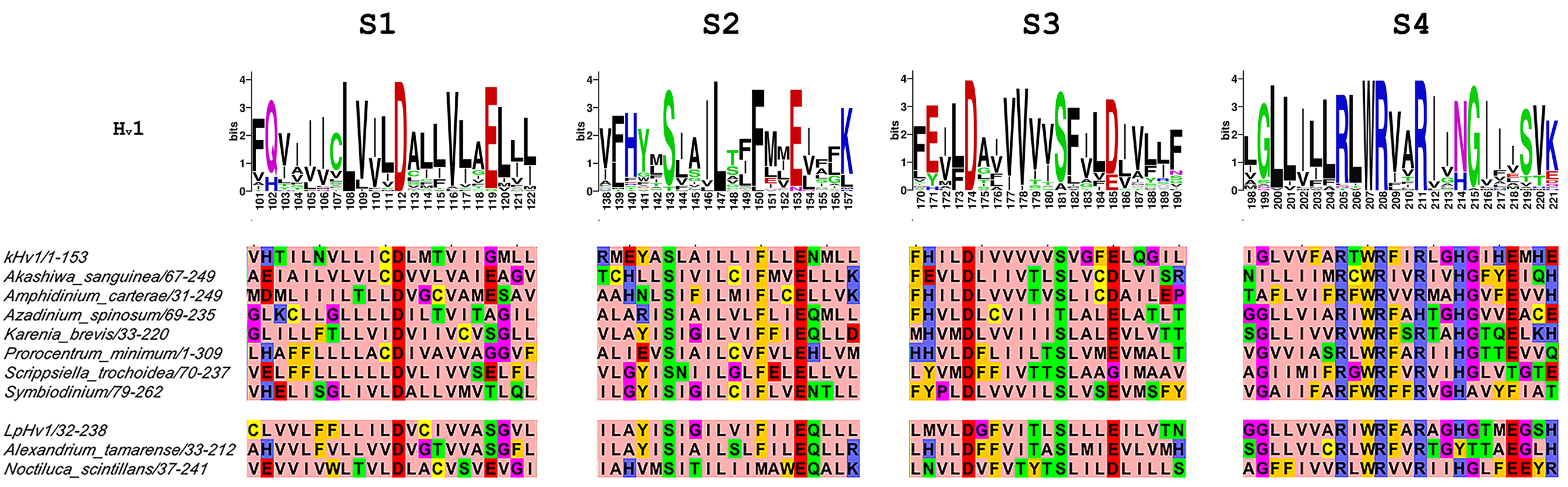

Supplement: S3 Fig — Sequences were found by BLAST searches of RNA-seq projects [35,36] and were aligned with MSA-Probs [72]. Dinoflagellate sequences are shown aligned with sequence logos of individual transmembrane helices obtained from an alignment of animal HV1s [12]. The sequence logos are numbered for hHV1. Overlapping partial sequences from Alexandrium monilatum are not shown. (TIF) [file pone.0171594.s006.tif]
